# Structural uterine changes in postpartum endometritis in cows

**DOI:** 10.14202/vetworld.2018.1473-1478

**Published:** 2018-10-22

**Authors:** S. M. Suleymanov, B. V. Usha, Yu. A. Vatnikov, E. D. Sotnikova, Eu. V. Kulikov, V. I. Parshina, M. V. Bolshakova, M. U. Lyshko, E. V. Romanova

**Affiliations:** 1 Department of Anatomy and Surgery, Voronezh State Agrarian University named after Emperor Peter I, Voronezh, Russia; 2 Department of Veterinary Medicine, Moscow State University of Food Production, Moscow, Russia; 3 Department of Veterinary Medicine, Agrarian Technological Institute, Peoples’ Friendship University of Russia (RUDN University), Moscow, Russia; 4 Department of Agrobiotechnology, Agrarian Technological Institute, Peoples’ Friendship University of Russia (RUDN University), Moscow, Russia

**Keywords:** cows, histological, macroscopic, postpartum endometritis, subclinical endometritis, ultrastructural changes of the endometrium

## Abstract

**Aim:**

The purpose of this work was to study the dynamics of structural manifestations of acute cases of postpartum endometritis in cows.

**Materials and Methods:**

The light and electron microscopy methods were used when studying structural changes in the endometrium in case of postpartum endometritis in seven cows. Sections of endometrial specimens for light microscopy, 5-7 µm thick, were stained with hematoxylin and eosin and also by Van Gieson’s. For electron microscopy, semi-thin sections were stained with Azur-2 in combination with basic fuchsin, as well as contrasting by lead citrate and uranyl acetate.

**Results:**

As a result of the study, we have established the following: Necrobiosis of the epithelial layer of the mucosa, cellular infiltration with shaped elements of blood in the functional layer, swelling of the cells of the uterine gland, and single microbial cells on the surface of the mucosa. We have noted edema of the stroma of the functional layer of the endometrium, swelling of the epithelial layer of the endometrial mucosa, and swelling of fibroblastic and lymphoid cells. Ultrastructural changes in endometrial cells in case of acute postpartum endometritis in cows are accompanied by the destruction of microvilli on the apical surface of the epithelium, an abundance of coccal microflora on the surface of the epithelium, necrobiosis of epithelial cells, and partial edema of the nucleus, and cytoplasm of the histiocyte.

**Conclusion:**

We had established that acute purulent-catarrhal dystrophic processes were observed in the structural organization of the endometrium. In the depth of catarrhal mucus on the surface of the endometrium, there was an abundance of bacterial flora, with diplococci being prevalent. In ultrastructural organization of the endometrium, we observed deep dystrophic and necrobiotic processes in the parenchyma and endometrial stroma, as well as exudative processes with a change in the integrity of the microcirculatory bed. Thus, to prevent an inflammatory process from turning into a latent form, it is necessary to detect acute postpartum endometritis promptly using diagnostic methods taking into account the obtained parameters of the dynamics of structural changes in the uterine tissues.

## Introduction

Many scientists believe that diseases of the reproductive organs in cattle represent one of the most important problems of modern veterinary obstetrics and gynecology since they are the main cause of long-term infertility in cows, accompanied by a decrease in milk productivity, cessation of lactation, and premature culling. In diseases of reproductive organs, the body and genitals develop profound structural changes in endometrium and myometrium characterized by hypotension of the uterus, swelling of the serous and muscular membranes, absence of signs of retraction, diffuse infiltration of the endometrium with lymphoid cell elements, necrosis, decay and rejection of the surface layer, severe vascularization, and blood filling of the blood vessels, as a result of which a bleeding wound surface is formed in the uterus and abundant bloody discharge from the genitals. Favorable conditions are created for penetration into the uterus cavity and reproduction of pathogenic microorganisms, the toxins of which intensify the metabolic disorders and microcirculation in the affected tissues. A vicious circle of pathological processes is established with a change in cause-effect relationship. In the development of the pathological process, the role of opportunistic microflora is significantly increased due to the increase in its virulence and the number of strains resistant to drugs, as well as to the high incidence of infection in pregnant animals.

Therefore, one of the central problems of insufficiently effective cows’ therapy and the risk of increasing postpartum complications is the constantly increasing drug resistance of pathogens. In this regard, continuous monitoring is necessary both over the composition of microorganisms and pathogens and the dynamics of development of their resistance. This allows us to develop an adequate strategy and tactics of antibiotic therapy in the specific conditions of each dairy complex or farm with the inclusion of a wide spectrum of action in the curative course.

Pathogenetic changes in endometritis in cows are largely determined by the state of natural body resistance while the level of local protection of any organ is inextricably linked to the level of its structural organization. This fact is not always taken into account in the pathogenesis of endometritis [1-13]. Along with this, pathology of the endometrium usually affects cows, which acquires subclinical mastitis in the 1^st^ day after calving. Studies have shown that 37.3% of cows in the postpartum period are affected by both mastitis and endometritis and, in 54% of cases, by purulent-catarrhal form [14], which is directly related to pathogenic and opportunistic microflora [15-21]. It should be noted that subclinical endometritis develops in case of untimely diagnosis or unskilled care, which is one of the most significant causes for reproductive system disorders in cattle breeding [22-28]. This disease is the most common of all uterine diseases and affects up to 30% of lactating dairy cows [29-31]. In individual farms, this figure ranges from 11% to 70% [32,33]. Statement of subclinical endometritis is manifested in multiple and ineffective inseminations. Along with this, rectal examination shows a lowered tone of the uterine horns in the preterm phase of the sexual cycle when the yellow body undergoes regression. At the end of the estrus, one can notice the presence of pus threads or flakes in the mucus [34]. A number of researchers indicate that in 24-64.7% of cows after clinical recovery, there are complications in the form of latent endometritis [35-37]. This can be easily missed when examining an animal. To prevent the process from turning into the latent form, it is necessary to detect acute postpartum endometritis promptly using diagnostic methods taking into account the dynamics of structural changes in the uterine tissues.

In this regard, many of the proposed drugs for the treatment of cows with endometritis, without regard to their effect on the structural organization of the uterus and the animal’s organism as a whole, do not have the desired effect or are far from inexpedient. Earlier, similar studies were conducted but without electron microscopy.

The aim of this work was to study the dynamics of structural manifestations of acute postpartum endometritis in cows using electron microscopy.

## Materials and Methods

### Ethical approval

This study obtained ethical clearance from the Bioethics Commission of Peoples’ Friendship University of Russia**.**

### Materials

The present study was carried out in accordance with the state program of All-Russian Scientific Research Veterinarian Institute for Pathology, Pharmacology, and Therapy in 2005-2015 in the Laboratory of Pathomorphology on the basis of commercial dairy farms in Voronezh Region. Experiments were conducted on a large number of livestock, and for selective research from experimental animals, the slaughter was carried out to take samples of the endometrium at meat control points. In addition, a biopsy material was used.

As a rule, the transition of the organism from the normal to the pathological state occurs at the level of the structural organization of the cells and tissues of organs in the absence of clinical deviations from the physiological norms of the organism. Thus, carrying out ultrastructural study is necessary to elucidate the pathogenetic mechanisms of the development of pathology in the endometrium in cows with a latent period in cows.

### Electron microscopy

When studying the structural changes in the endometrium in postpartum endometritis in seven cows, we used the light and electron microscopy as the most reliable method for diagnosing acute postpartum endometritis [38].

After an autopsy of cows euthanized for household needs and evaluation of the macroscopic picture indicating purulent endometritis, the material for histology was fixed in 10% solution of neutral formalin and Carnoy fluid, and sections 5-7 µm thick were stained with hematoxylin-eosin, Van Gieson’s, and Azur-2 in combination with basic fuchsin and toluidine blue. Fixation of the material for electron microscopy was carried out in the 2.5% solution of glutaraldehyde with postfixation in 1% solution of osmium tetroxide. The material was encapsulated in Epon 812. Semi-thin sections stained with Azur-2 in combination with the basic fuchsin were analyzed in the light microscope “Leica.” Ultrathin sections were prepared on Ultramicrotome Ultracut “Leica,” contrasted with lead citrate and uranyl acetate, and analyzed in Philips EM 208 electronic microscope.

## Results

**Figure-1:**
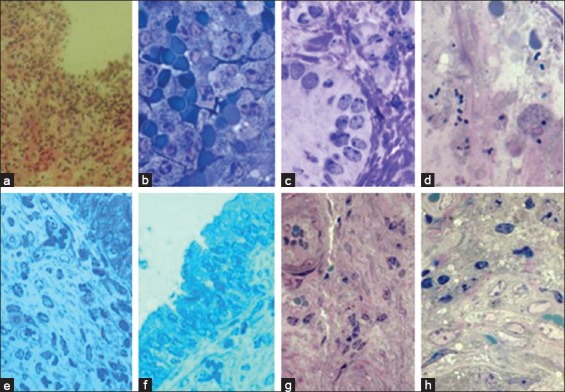
Histological changes in the mucous membrane of the uterus in case of acute postpartum purulent-catarrhal endometritis in cows: (a) Necrobiosis of the epithelial layer of the mucosa, (b) cellular infiltration with the shaped blood elements in the functional layer, (c) swelling of the cells of the uterine gland, (d) single microbes on the mucosa, (e) swelling of the stroma of the functional layer of the endometrium, (f) swelling of the epithelial layer of the endometrial mucosa, (g) endothelial dystrophy of vessels, and (h) swelling of fibroblastic and lymphoid cells. Color: (a) Hematoxylin-eosin, (b-d, g, h) Azur-2 in combination with basic fuchsin, and (e and f) toluidine blue. Magnification: (a) 320, (b-d and e) 800, (f) 700, (g) 400, and (h) 200.

At autopsy revealed hyperemia and puffiness of the mucous membrane of the vaginal vestibule, as well as abrasions, spot, and banded hemorrhages. In the lumen of the vagina, there was a purulent-mucous exudate, which was released from the cervical canal. Cervical canal was opened. Folds of the vaginal part of the cervix appeared swollen, edematous, and hyperemic. Point and banded hemorrhages were also detected here. In some places, there was the complete absence of epithelial lining, while in the structural organization of the functional layer of the mucous membrane, there was an abundance of blood cells against the background of lymphoid and epithelial clusters. An endometrium appeared swollen, abundantly infiltrated by the emigrated cells. In the thickness of the endometrium, the uterine glands were edematous and contained swollen epithelial cells. They were surrounded by inflammatory infiltrates, often exudative. Epithelium lining of the inner surface of the uterine glands did not preserve its integrity. The nuclei of cells of the glandular epithelium were found predominantly in the middle part, in places where there was a decrease in nuclei number and their wrinkling. Deeper lesions of the uterine endometrial glands were accompanied by necrobiosis of glandular epithelium cells and their rejection from the stroma of the uterine gland in its lumen. In the surface layer of the endometrium, there was cellular detritus with an inflammatory infiltrate. An abundant catarrhal exudate was accumulated here with shaped blood elements and microbial cells. The uterine vascular bed was characterized by swelling of vascular endothelial. Furthermore, there was a significant change to smooth muscle cells, fibroblasts, and other tissue elements of the endometrium. In the deep layers of the endometrium, the uterine glands were mostly found in a state of necrobiosis (Figure-1).

**Figure-2:**
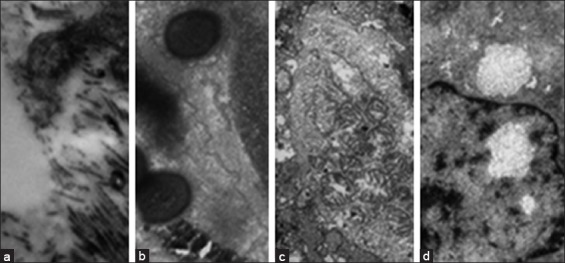
Ultrastructural changes in endometrial cells in acute postpartum endometritis in cows: (a) Destruction of microvilli on the apical surface of the epithelium, (b) abundance of coccal microflora on the surface of the epithelium, (c) necrobiosis of epithelial cells, (d) partial edema of the nucleus and cytoplasm of the histiocyte. Magnification: (a) 3500, (b) 5600, and (c and d) 2800.

Electron microscopy on the apical part of the epithelium of the endometrial mucosa revealed the destruction of microvilli. Here, we detected microflora and catarrhal mucus with accumulated microflora. In the ultrastructural organization of the endometrium, deep dystrophic and necrobiotic processes in the parenchyma and stromal cells, as well as exudative processes with a change in the integrity of the microvasculature, were observed (Figure-2).

In the cellular infiltration of the bacterial flora, diplococci with deformed membranes prevailed. We revealed the violation of microbial cell function against the background of histiocytic macrophages. There were increased numbers of apoptotic bodies and necrobiotic cells in the uterus mucosa. There was partial swelling of the nucleus and cytoplasm in histiocytes of the endometrium. There were fragments of plasma cells and lymphocytes, and macrophage cells in the uterine mucosa were activated. Swelling of the wall of the blood capillaries and their endothelium was noted. An integrity of the microcirculatory vascular bed was disturbed.

## Discussion

Acute postpartum endometritis in cows is characterized by catarrhal discharge from the uterus, mucopurulent outflow from it. Cervical canal during this period, as a rule, is open. In this case, in cows on 10-15 days after calving, involution of the uterus is significantly delayed, and its magnitude varies within the range of 35-55±5-7 cm, which corresponds to its value in the period of 3-4 months of pregnancy [39]. Catarrhal exudate accumulates mainly around and near the caruncles [40] and against the background of a mucous catarrhal deposit on the mucosa where there are multiple points and spotted hemorrhages. At the level of light microscopy in the mucous membrane of the uterus, there is a violation of the integrity of integumentary epithelium, its necrosis, and desquamation. Polymorphonuclear cells are visible at least of all [25]. These exudative-infiltrative processes encompass the entire thickness of the endometrium and characterize its edema [40]. Therefore, the endometrium appears swollen, abundantly infiltrated by various cells. Uterine glands in the thickness of the endometrium, as a rule, are swollen and contain swollen cells in the environment of inflammatory-exudative infiltrates. Epithelium lining the inner surface of the uterine glands does not retain its integrity. Nuclei of cells of the glandular epithelium are located mainly in the middle part, in places, there is a decrease in nuclei in size and their pycnosis [41,42]. Deeper lesions of the uterine glands of the endometrium are accompanied by necrobiosis of the cells of the glandular epithelium and their rejection from the stroma of the uterine gland into its lumen. Functional characteristics of the revealed histomorphologic changes in the endometrium in cows with acute postpartum purulent catarrhal endometrium are refined by electron microscopy, in the form of ultrastructural organization of mucosal cells. On the apical part of the epithelium, which remains intact on the surface of the endometrial mucosa, destruction of microvilli and accumulation of catarrhal mucus with microflora are noted, which herald functional disturbance of endometrial cells. Furthermore, an apical part of the epithelium is destroyed, loses microvilli, vacuolizes, and becomes a homogeneous mass. In the thick of catarrhal mucus on the surface of the endometrium, there is an abundance of bacterial flora, in which the prevalence of diplococci is observed. In places, there is destruction of the bacterial flora and the appearance of macrophages and histiocytes. However, there are single bacteria against the background of multiple enlightened mitochondria in the epithelial layer and deep layers of the endometrium. In most cases, necrotic processes are observed. In the endometrium, microcirculatory bed in the parenchyma of the organ significantly changes. In this case, sharp swelling of the endothelium of blood capillaries, vessels, and sometimes even small capillaries is observed.

## Conclusion

Macroscopic changes in postpartum endometritis in cows are characterized by the presence of hyperemia and swelling on the mucous membrane of the vaginal vestibule, vagina, and cervix with spot-spotted and banded hemorrhages covered with purulent mucous exudate. Abundant catarrhal deposits with multiple spot-spotted hemorrhages can also be observed. At days 10-15 after calving, the size of the uterus in cows varies within the range of 35-55±5-7 cm. At the level of light microscopy in the mucous membrane of the endometrium, in case of postpartum endometritis of cows, we can see the violation of the integrity of integumentary epithelium, followed by its disintegration and desquamation into the uterine cavity. Pathological process is penetrating the deeper layers of endo- and myometrium in the form of exudative-infiltrative changes. Edema of the functional layer of endometrium is accompanied by diffuse cell infiltration containing an abundance of necrobiotic leukocytes, lymphoid, and tissue cells as well as accumulation of purulent cells in the lumen of the uterine glands. Catarrhal exudate predominates on the exposed surface of the endometrial mucosa. Catarrhal exudate comprises loose epithelium, blood cells, and colonies of microorganisms. In the ultrastructure of endometrial cells, there are irreversible changes in cytoplasmic organelles, nuclear karyoplasm, and nucleoli. Diplococci with deformed membranes are prevalent in the cellular infiltration of the bacterial flora. We can see the violation of microbial cell function against the background of histiocytic macrophages. The number of apoptotic bodies and necrobiotic cells in the functional layer of the endometrium is increased. There is a partial swelling of nuclei and cytoplasm in histiocytes of the endometrium. There are fragments of plasmatic cells and lymphocytes, and macrophage cells are activated in the functional layer of the endometrium. Swelling of the walls of blood capillaries and endothelium occurs. The integrity of the microcirculatory bed is disturbed. To minimize the influence of subclinical endometritis on the fertility of dairy cows, it is first necessary to know the causes and predisposing factors of subclinical endometritis [39] and also to detect and treat acute postpartum endometritis promptly as one of the factors in the development of the subclinical form of the disease.

## Authors’ Contributions

SMS, BVU, YAV, and EVK had the original idea for the study and carried out the design. MVB collected the samples. EVK, VIP, EVR, and MUL were responsible for data analysis, data cleaning. YAV, VIP, EVK drafted the manuscript. The final draft manuscript was revised by all authors. All authors read and approved the final manuscript.
